# Evidence for T Cell Help in the IgG Response against Tandemly Repetitive *Trypanosoma cruzi* B13 Protein in Chronic Chagas Disease Patients

**DOI:** 10.1155/2012/635873

**Published:** 2012-02-19

**Authors:** Marcia Duranti, Ludmila Camargo, Gabriel Victora, Barbara Ianni, Paula Buck, Charles Mady, Jorge Kalil, Bianca Zingales, Edecio Cunha-Neto

**Affiliations:** ^1^Laboratory of Immunology, Heart Institute (InCor), School of Medicine, University of São Paulo, 05403-900 São Paulo, SP, Brazil; ^2^Departamento de Bioquímica, Instituto de Química, Universidade de São Paulo, 05508-800 São Paulo, SP, Brazil; ^3^Division of Clinical Immunology and Allergy, School of Medicine, University of São Paulo, 01246-903 São Paulo, SP, Brazil; ^4^Institute for Investigation in Immunology (iii), INCT, 05403-900 São Paulo, SP, Brazil; ^5^Whitehead Institute for Biomedical Research, Nine Cambridge Center, Cambridge, MA 02142, USA; ^6^Myocardiopathies Unit, Heart Institute (InCor), School of Medicine, University of São Paulo, 05403-900 São Paulo, SP, Brazil

## Abstract

The tandemly repetitive *Trypanosoma cruzi* B13 protein is an immunodominant antigen among Chagas disease patients. Such repetitive domains may behave as T-independent antigens. However, T cells can recognize B13 epitopes in an HLA class II-restricted fashion and could potentially provide cognate T cell help and boost antibody titers. We assessed whether the presence of HLA class II molecules able to present B13 epitopes to T cells could affect anti-B13 IgG levels in a cognate fashion, in both major clinical forms of chronic Chagas disease. We found no difference between anti-B13 IgG antibody levels between patients carrying HLA class II molecules associated to T cell responses or other alleles. The predominant anti-B13 IgG subclass was IgG1, with negligible IgG2, suggesting a T-dependent, noncognate help for antibody production. In addition, the finding of increased anti-B13 IgG levels in sera from CCC patients indicates that clinical presentation is associated with increased anti-B13 antibody levels.

## 1. Introduction

Pathogenic protozoa like *Trypanosoma cruzi*, the causative agent of Chagas' disease, contain regions of tandemly repetitive amino acid sequences embedded in several immunodominant protein antigens [1]. The function of this type of protein domains is poorly understood. The repetitive elements are diverse in number, size, and distribution with several common features like their immunodominance, the bias in the component aminoacids, and an unusual evolutionary history [2]. Little is known about the molecular mechanisms that lead to the immunodominance of repeated sequences, except for their multivalency which can make them activate B cells directly and behave, in some cases, as T-independent antigens by crosslinking hapten-specific surface immunoglobulin [3]. It is known that the repetitiveness of a variety of agents causes MHC class II-independent, T cell independent activation of B cells, and it has been shown that tandemly repetitive carbohydrate antigens elicit IgG2 subclass antibody responses in humans [4–6]. B13 protein is an immunodominant recombinant antigen which encodes part of the tandemly repeated domain of the *T. cruzi* 140/116 kDa protein located on the surface of infective trypomastigotes from several strains. B13 protein from the Y strain of *T. cruzi* is formed by 19 tandemly repeated degenerate copies of a 12-amino acid motif P(L)P(S,A)P(L)FGQAAA(E)G(A)D(G)K, where residues in parentheses replace the preceding residue in different repeats [7]. B13 protein is recognized by IgG serum antibodies from 97% of *T. cruzi *infected individuals in Latin America, bearing both forms of chronic Chagas' disease: chronic Chagas' disease cardiomyopathy (CCC) and the asymptomatic/indeterminate form (ASY) [8, 9]. Indeed, there is evidence supporting that the antibody response towards repetitive protozoan antigens is T cell-independent [10, 11].

However, it cannot be excluded that T cell help could boost antibody titers and promote IgG subclass switch. Cognate T cell help is provided when T cells and B cells of the same antigen specificity are in close contact, and wherein the antigen is presented directly to T cells in association with B cell surface HLA class II molecules. Cognate T cell help occurs only in individuals who carry HLA class II molecules that allow presentation and recognition of that antigen to T cells. Noncognate T cell help, on the other hand, occurs when T cells activated by one antigen presented by an antigen-presenting cell provide help for antibody production to a B cell with different antigen specificity [12, 13]. Repetitive sequences in protozoan parasite antigens have been shown to elicit T cell-dependent immune responses. It has been reported that peripheral T cells from *P. falciparum *malaria patients recognize T cell epitopes in EB200, a repetitive region of the *P. falciparum *antigen Pf332, the Pf155 repetitive domain of ring-infected erythrocyte surface antigen (RESA) and the repetitive domain of circumsporozoite CS protein [14, 15]. We have previously shown that PBMC proliferative and cytokine responses to B13 and its synthetic peptides are frequent among *T. cruzi*-infected subjects [8, 16]. We also found that T cell responses to B13 protein are restricted to patients carrying certain HLA class II alleles (HLA-DQA1*0501(DQ7), HLA-DR1, and HLA-DR2), which bind and present B13 peptides directly [8]. It is thus conceivable that B13-specific T cells could provide cognate help for the production of anti-B13 antibodies among *T. cruzi-*infected patients that carry such HLA class II molecules. To test this hypothesis, we will assess whether the presence of at least one of these HLA alleles increases the magnitude of IgG responses to the B13 antigen in Chagas patients stratified into ASY or CCC forms of disease. We will also assess whether the presence of B13-presenting HLA alleles would influence the IgG subclass profile, since distinct IgG subclass profiles have been associated to T-dependent or T-independent IgG responses [17, 18].

## 2. Methods

### 2.1. Study Population

Serum samples were taken from a total of 112 patients infected with *T. cruzi*, stratified into the two forms of Chagas' disease: chronic Chagas cardiomyopathy (CCC; *n* = 72) and the indeterminate/asymptomatic form (ASY; *n* = 40). The patients were recruited from the Heart Institute (InCor), University of Sao Paulo School of Medicine. Chronic Chagas cardiomyopathy (CCC) subjects fulfilled the following diagnostic criteria: positive *T. cruzi* serology, typical ECG abnormalities (left anterior hemiblock and/or right bundle branch hemiblock), with or without varying degrees of ventricular dysfunction, with all other etiologies of ventricular dysfunction/heart failure excluded. Indeterminate/asymptomatic subjects (ASY) showed positive *T. cruzi* serology but normal ECG and bidimensional echocardiography. The study protocol was approved by the Institutional Review Board of the University of São Paulo School of Medicine. All patients had given informed consent for the use of their blood samples for research.

### 2.2. HLA Class II Typing

DNA was extracted by either DTAB/CTAB or salting out methods [19]. DR typing was performed by low-resolution PCR-SSP as previously described [20–22]. DQA1, and DQB1 typing was performed by PCR-SSO using generic primers for exon-2 amplification [23].

### 2.3. Direct ELISA

96-well polystyrene plates (Corning, USA) were coated with 30 ng/well of recombinant B13 protein in carbonate-bicarbonate buffer, pH 9.6, for 16 h at 4°C. Plates were washed with PBS and blocked with PBS-2% BSA for 30′ at 37°C. Serum samples at 1/50 dilution were incubated for 1 h at 37°C. After ten washing cycles with PBS-0.1% Tween 20 (PBS-T), wells were incubated with HRP-conjugated anti-human IgG for 30′ at 37°C. After ten additional washing cycles with PBS-T, samples were incubated with substrate solution (4 mg OPD in 10 mL citrate buffer, pH 5.0) for 30′ at 37°C. Absorbance at 490 nm wavelength was measured using an automated plate spectrophotometer.

### 2.4. IgG Subclass Measurement

Anti-B13 antibody subclass measurement was carried out by ELISA following essentially the same protocol described above except that a concentration curve was made by incubating different concentrations of IgG subclass (IgG1, 2, 3, and 4) standards. We also used HRP conjugated mouse anti-human IgG1, 2, 3, and 4 (Pharminrgen BD) diluted in PBS containing 1% calf serum PBS-T that was added to each well (Dilution of 1 : 1,000). After 1-hr incubation at 37°C, plates were incubated with substrate solution (4 mg OPD in 10 mL citrate buffer, pH 5.0) for 30′ at 37°C. Absorbance at 490 nm wavelength was measured using an automated plate spectrophotometer. The cutoff value was determined as the mean value of optical densities from control sera plus 3 standard deviations.

## 3. Statistical Analysis

Groups were compared by a nonparametrical test (Mann-Whitney Rank Sum Test) with GraphPad InStat software (version 5.0; GraphPad). Results were expressed as medians and interquartile ranges. *P*-values were considered significant if <0.05.

## 4. Results

### 4.1. Anti-B13 IgG Antibodies among Carriers and Noncarriers of B13-Binding HLA Class II Molecules

**Figure 1:**
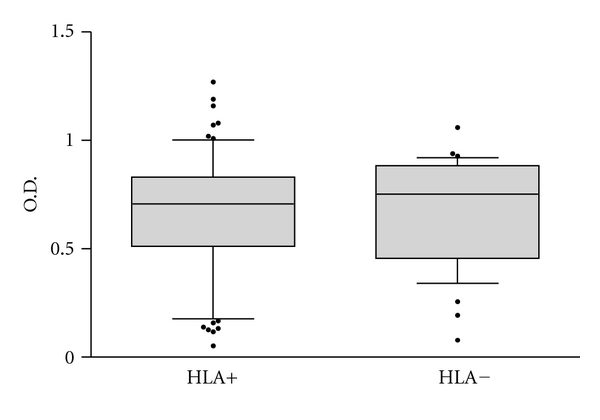
Direct ELISA for total IgG reactivity to recombinant B13 protein in the solid phase. 76 subjects bearing HLA alleles capable of presenting B13 peptides to T cells HLA-DQ7, DR1, and DR2 (HLA+), and 36 subjects bearing other HLA alleles (HLA−). The horizontal bar indicates median absorbance in each group. *P* = 1.00, *Mann-Whitney Rank Sum Test*.

**Figure 2:**
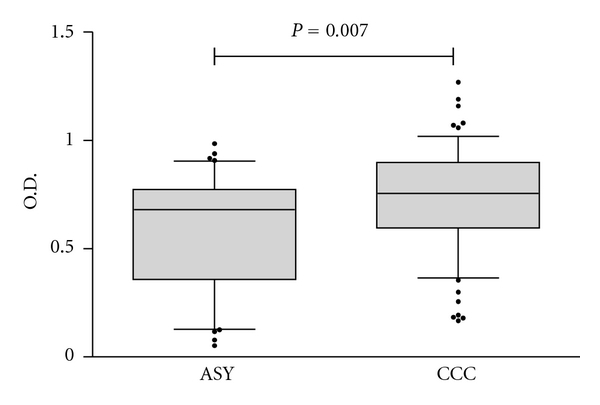
Direct ELISA for total IgG reactivity to recombinant B13 protein in the solid phase. Comparison between CCC (subjects with chronic Chagas Cardiomyopathy) and ASY (subjects with indeterminate/asymptomatic form of the disease). ASY and CCC subjects (median Abs. = 0.756 versus 0.681, resp.). The box plots whiskers show median values and 25–75% percentile; the 10% and 90% percentile; ou tlier points are shown above and below the figure.

**Figure 3:**
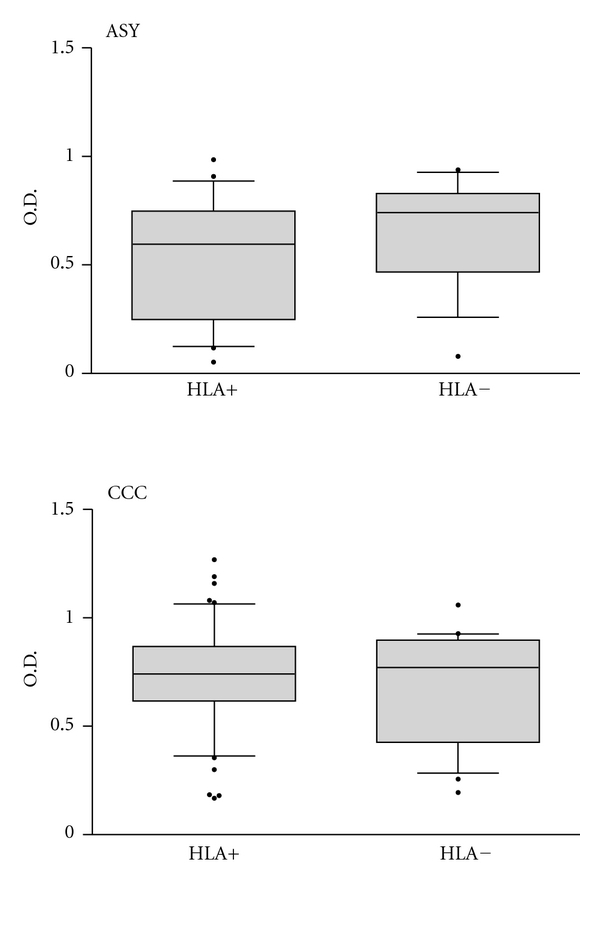
Direct ELISA for total IgG reactivity to recombinant B13 protein in the solid phase. The HLA+ group (subjects bearing HLA alleles capable of presenting B13 peptides to T cells,HLA-DQ7, DR1, and DR2); and the HLA− group (subjects bearing other HLA alleles). The horizontal bar indicates median absorbance in each group.

We divided the 112 *T. cruzi*-infected patients into two groups, 76 subjects bearing one or more of the HLA alleles required for presentation of B13 to T cells (HLA-DQ7, DR1, and DR2), termed HLA+, and 36 subjects lacking these alleles, HLA−.  Figure 1 shows that there was no significant difference in total IgG reactivity to B13 between HLA+ and HLA− patients. We then subdivided the group of subjects into different clinical presentations of Chagas disease, CCC (*n* = 72) and ASY (*n* = 40). Sera from CCC patients displayed a significantly higher total IgG anti-B13 reactivity than ASY patients, although there was some overlap between samples in both groups (Figure 2). When we further stratified the clinical groups in HLA+ and HLA− patients, we observed no significant difference in total IgG anti-B13 reactivity between samples from HLA+ to HLA− patients from the CCC and ASY groups (Figure 3).

### 4.2. Anti-B13 IgG Subclass Determination among Carriers and Noncarriers of B13-Binding HLA Class II Molecules

**Figure 4:**
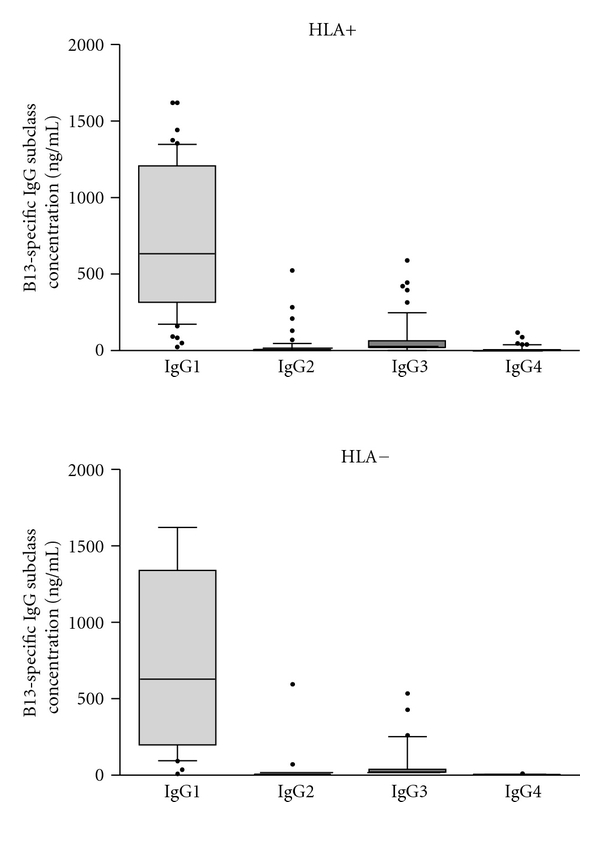
Direct ELISA for levels of the four subclasses of IgG reactive to recombinant B13 protein in the solid phase in HLA+ subjects (*n* = 53) and HLA− subjects (*n* = 34). The horizontal bar indicates median concentration of each IgG subclass in each group: IgG1; IgG2; IgG3; IgG4.

**Figure 5:**
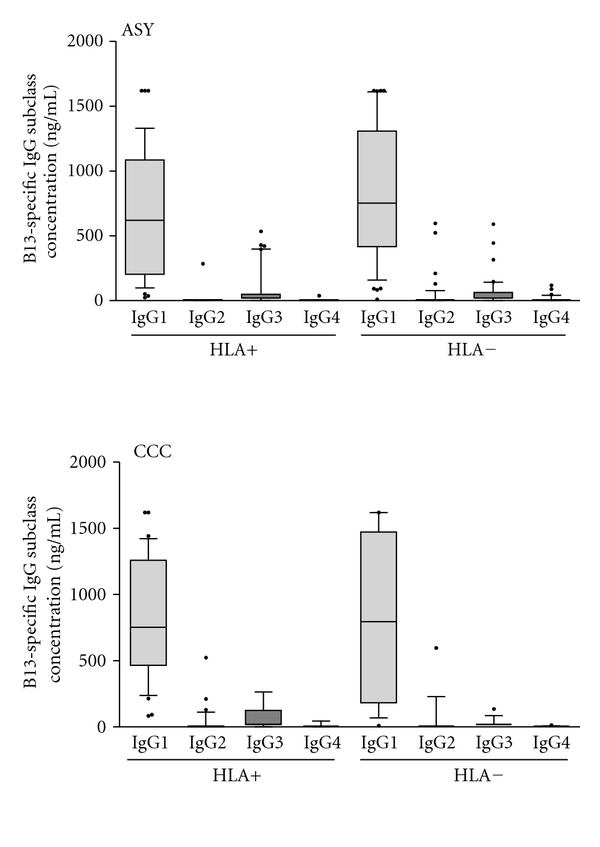
Direct ELISA for levels of the four subclasses of IgG reactive to recombinant B13 protein in the solid phase in ASY and CCC subjects; HLA+: subjects bearing HLA alleles capable of presenting B13 peptides to T cells (HLA-DQ7, DR1, and DR2); HLA−: subjects bearing other HLA alleles. The horizontal bar indicates median concentration of each subclass in each group: IgG1; 2: IgG2; 3: IgG3; 4: IgG4.

We evaluated whether the presence or absence of the relevant HLA alleles was associated with differences in distribution among the four IgG subclasses analyzing 87 subjects (HLA+; *n* = 53, and HLA−; *n* = 34).  Figure 4 shows that there were no differences in the distribution of IgG subclasses between groups, with IgG subclass levels ranking from IgG1 > IgG3 > IgG2 > IgG4 in both HLA+ and HLA− subjects. We further stratified the subjects in our study separating them into the two clinical forms of Chagas disease, CCC (*n* = 32) and ASY (*n* = 39), inside the two HLA groups (HLA+ and HLA−), but no significant difference was observed (Figure 5).

## 5. Discussion

In this study, we further analyzed the magnitude of IgG responses and IgG subclasses to the *T. cruzi* tandemly repetitive B13 antigen in Chagas patients. We demonstrated that there is no difference in anti-B13 IgG antibody levels between patients carrying the B13 presenting HLA class II molecules, associated to T cell responses (HLA+) and those carrying other alleles (HLA−). Nevertheless, we found increased anti-B13 IgG levels in sera from CCC as compared to ASY patients, but no differences in anti-B13 IgG levels were observed between HLA+ and HLA− patients in the CCC and ASY groups. Regarding IgG subclasses, there was a predominance of IgG1 against B13 protein antigen, in both patient groups (HLA+ and HLA−); we also found no difference in levels of any of the IgG subtypes in the CCC and ASY groups, regardless of their HLA type.

The finding that anti-B13 IgG antibody levels of patients carrying the B13-presenting HLA class II molecules, associated to T cell responses, are similar to those carrying other alleles argues against a role for genetically restricted (thus, cognate) T cell help to anti-B13 antibody production. On the other hand, the IgG subclass distribution of anti-B13 antibodies—predominantly IgG1—argues against its being a strict T-independent (T1-2) antigen, since, even though it shows no genetic restriction, it fails to induce IgG2 antibodies as descript for tandemly repetitive carbohydrate antigens [4–6]. Antibodies against nonrepetitive (and presumably T-dependent) recombinant proteins [24] or *T. cruzi* epimastigote homogenate [25] in sera from chronic Chagasic patients were essentially of the IgG1 and IgG3 subclasses. This established the IgG1/IgG3 profile as the standard response against nonrepetitive *T. cruzi* proteins. Our finding that the predominant IgG subclass in the anti-B13 response is IgG1 supports the idea that the IgG antibodies may be generated under noncognate T cell help. This is further supported by the absence of genetic restriction of the anti-B13 IgG antibody responses. It is conceivable that B13-specific B cells may receive noncognate help from T cells specific for other *T. cruzi* proteins, cointernalized with B13, during *T. cruzi* infection. The same mechanism has been observed in systemic lupus erythematosus, where DNA-specific B cells may be helped by T cells specific for histones or other nuclear proteins [26]. However, since the nature of the B13 protein available for endocytosis by B cells during *T. cruzi* infection is unknown, this argument remains speculative.

The significant difference found in anti-B13 IgG antibody levels in CCC as compared to ASY is striking. This has also been reported for a few other recombinant *T. cruzi* antigens, such as antigens JL5, JL9, and the C-terminal region of *T. cruzi* HSP70 [27]. B13 and JL5 have been described as cross-reactive with cardiac myosin [28–30] and cardiovascular beta-adrenergic and cholinergic receptors respectively [31]. It is possible that the higher levels displayed in sera from CCC patients of these two potentially pathogenic cross-reactive antibody systems are biologically relevant in the generation of pathological autoimmunity. Our results indicate that, even though B13 protein is a tandemly repetitive, TI antigen, the detectable IgG response to it apparently depends on noncognate T cell help. Further studies, including assessing direct B-cell activation by B13, may be necessary to definitively establish the nature of the serum IgG response to B13 found in chronic Chagas disease patients.
